# Web Search Engine Misinformation Notifier Extension (SEMiNExt): A Machine Learning Based Approach during COVID-19 Pandemic

**DOI:** 10.3390/healthcare9020156

**Published:** 2021-02-03

**Authors:** Abdullah Bin Shams, Ehsanul Hoque Apu, Ashiqur Rahman, Md. Mohsin Sarker Raihan, Nazeeba Siddika, Rahat Bin Preo, Molla Rashied Hussein, Shabnam Mostari, Russell Kabir

**Affiliations:** 1 The Edward S. Rogers Sr. Department of Electrical & Computer Engineering, University of Toronto, Toronto, ON M5S 3G4, Canada; 2 Institute of Quantitative Health Science, Department of Biomedical Engineering, Michigan State University, East Lansing, MI 48824, USA; 3 The Intervention Centre, Oslo University Hospital, 0372 Oslo, Norway; 4 Department of Computer Science, Northern Illinois University, DeKalb, IL 60115-2828, USA; ashiqur.r@niu.edu; 5 Department of Biomedical Engineering, Khulna University of Engineering & Technology (KUET), Khulna 9203, Bangladesh; msr.raihan@gmail.com; 6 Center for Environmental and Respiratory Health Research (CERH), Faculty of Medicine, University of Oulu, 90014 Oulu, Finland; nazeeba.siddika@oulu.fi; 7 Department of Electrical and Electronic Engineering, Bangladesh University of Business and Technology, Dhaka 1216, Bangladesh; rahatpreo1004@gmail.com; 8 Department of Computer Science and Engineering, University of Asia Pacific, Dhaka 1205, Bangladesh; mrh.cse@uap-bd.edu; 9 Aspire to Innovate (a2i) Programme, ICT Division, Dhaka 1207, Bangladesh; shabnam.mostari@a2i.gov.bd; 10 School of Allied Health, Faculty of Health, Education , Medicine and Social Care, Anglia Ruskin University, Chelmsford CM1 1SQ, UK; russell.kabir@anglia.ac.uk

**Keywords:** COVID-19, public health misinformation, web search engine, notifier extension, natural language processing, machine learning, artificial neural network

## Abstract

Misinformation such as on coronavirus disease 2019 (COVID-19) drugs, vaccination or presentation of its treatment from untrusted sources have shown dramatic consequences on public health. Authorities have deployed several surveillance tools to detect and slow down the rapid misinformation spread online. Large quantities of unverified information are available online and at present there is no real-time tool available to alert a user about false information during online health inquiries over a web search engine. To bridge this gap, we propose a web search engine misinformation notifier extension (SEMiNExt). Natural language processing (NLP) and machine learning algorithm have been successfully integrated into the extension. This enables SEMiNExt to read the user query from the search bar, classify the veracity of the query and notify the authenticity of the query to the user, all in real-time to prevent the spread of misinformation. Our results show that SEMiNExt under artificial neural network (ANN) works best with an accuracy of 93%, *F*1-score of 92%, precision of 92% and a recall of 93% when 80% of the data is trained. Moreover, ANN is able to predict with a very high accuracy even for a small training data size. This is very important for an early detection of new misinformation from a small data sample available online that can significantly reduce the spread of misinformation and maximize public health safety. The SEMiNExt approach has introduced the possibility to improve online health management system by showing misinformation notifications in real-time, enabling safer web-based searching on health-related issues.

## 1. Introduction

The current internet-based smart technological advancements, such as wearable technology and mobile phone applications (apps), have enabled constant real-time health monitoring of people in various diseases, and users have become more conscious regarding their health and about that of their loved ones [1]. Even long before the COVID-19 pandemic, in 2012, researchers reported that almost 59% of US adults searched for health information online. However, the number increased to 75% more recently, with more than a billion health-related searches occurring on Google search engines daily. There is no doubt that individuals are relying more on internet search engines regarding their health-related queries [2]. 

Misinformation is fake, unreliable, or not scientifically validated written material regardless of intentional authorship. Most misinformation-oriented discussions have been focused on venomous acts to taint the social media platforms with harmful and inaccurate information. False or fake news, misinterpretation of a drug protocol, or the presentation of unrealistic claims have dramatic effects on public health [3]. It draws attention away from scientific fact and real public health challenges and misleads healthcare professionals [3]. The flow of biased and inaccurate information can quickly dilute the seriousness of the actual issue and hamper the functioning of healthcare policy and disaster management [4]. 

Misinformation was widespread during the primary stages of the human immunodeficiency viruses (HIV) epidemic. It was also plagued by conspiracy theories, rumors, and misinformation for many years, with the effects still visible in regions to this day [5]. At the time of the avian influenza H5N1 outbreak in 2004, the World Health Organization’s (WHO) Western Pacific Regional Office identified around forty rumors. Among them, nine were verified to be accurate [6]. During the West African Ebola virus epidemic in 2014, there was widespread fear and attention among the United States-based users, followers of Western media, and social media platforms such as Twitter [7]. 

The current ongoing coronavirus disease 2019 (COVID-19) pandemic situation has potentially affected the capacity of health facilities, even in developed countries where there are proven and robust healthcare systems [8,9]. The pandemic has brought unexpected, sudden, and unparalleled damages, changes to global health and socio-economic frameworks. For minimizing COVID-19 spread, most countries have enforced a societal-level lockdown, and citizens have resumed offices remotely and online activities by staying in residences as much as possible [10]. During the lockdown, people are using internet search engines and social media platforms to gain information about COVID-19. The nature of social media impact varies depending on an individual’s gender, age, and level of education. Social media has played a vital role in spreading anxiety about COVID-19 in many territories. The COVID-19 pandemic has been termed as the first social media infodemic [11]. At the Italian lockdown period, even near bedtime, people increased the usage of digital media and internet usage [12]. In an Italian CoMuNe laboratory, Gallotti et al. have set up a COVID-19 “infodemic observatory,” where they used artificial intelligence (AI)-integrated automated software to follow 4.7 million tweets on COVID-19 streaming past every day [13]. On the other hand, Cinelli et al. reported about 1.3 million posts and 7.5 million comments on COVID-19 from several social media platforms [12]. 

Pandemic fear among the population can promote online searches for unproven and unprescribed therapies. If fake news and misinformation spread in an uncontrolled manner, it may become fatal [14]. Moreover, the poisoning of over two thousand Iranians by swallowing methanol took place since a misleading social media message urged the mass people in Iran to prevent the SARS-CoV-2 infection by drinking alcohol. Around nine hundred illicit alcohol poisoned patients had to get admitted to intensive care unit (ICU) with a fatality of almost three hundred unfortunate deaths [15]. Global coverage about panic-buying in the online media and social networks only served to promote the same behavior, which caused stockpiling of drugs and vaccines as a method of preparation in the COVID-19 pandemic [16]. 

False news generally travels faster than reliable and authentic reports on social media platforms such as Twitter [17,18]. Much misinformation and rumor spread around online search engines and social platforms regarding COVID-19. Technology entrepreneurs and investors shared a document on Twitter, promoting the malaria drug chloroquine for treating COVID-19. Many mentioned successful therapeutic outcomes in China and South Korea. Despite that, when high profile individuals, such as entrepreneur Elon Musk, promoted chloroquine, it attracted the attention of the general population, which could lead to personal decision-making about treatment options [17,19]. Misinformation circulated rapidly before the results of a small, non-randomized French trial of the related drug hydroxychloroquine; at that time the article was in press [20]. Hospitals have reported poisoning cases, where individuals suffered from toxicity from chloroquine containing pills, which they intended to take for COVID-19 [19]. During early July 2020, the WHO released a press note about discontinuing hydroxychloroquine. In the most recently published original article, it has been shown that hydroxychloroquine did not improve clinical status at two weeks, compared with standard care [21]. 

To control and track the COVID-19 trajectory, different governments have implemented various digital health surveillance tools, such as smartphone-based apps for COVID-19 contact tracing [22]. Researchers have suggested that the US Food and Drug Administration (FDA) must warn the general population against collecting unapproved, unprescribed therapies and medicines [17]. Google integrated an educational website into search results related to the COVID-19 outbreak, and they mentioned that it could be extended for unapproved COVID-19 medication-related searches [17]. The rise in the use of social media spreads misinformation and unproven treatments about infectious diseases such as COVID-19 at lightning speed. An infinite amount of misinformation is available over the internet and it keeps rising day by day. Therefore, machine learning (ML) is one of the potential candidates as a counterstrategy to handle and classify the authenticity of a large volume of information automatically and in real-time. To identify fake news, scientists have developed ML-based false news and misinformation credibility inference models, which form a deep diffusive network model to memorize news articles, writers, and topics [23,24]. 

To combat the propagation of misinformation, some of the biggest internet and social media companies have introduced technical gatekeepers to control what information can be sent over their networks. WhatsApp launched a chatbot to connect its millions of users with various fact-checking organizations across the globe [25]. This allows a user to double-check the information. The platform also introduced the WHO’s alert notification. This service responds to public queries about Coronavirus and provides official information 24 h a day, worldwide [26]. Facebook deployed machine learning algorithms to detect the advertisement of false claims such as homeopathic remedies that can prevent, cure, or protect against COVID-19 [27]. It also bans the sale of commercial safety products during the outbreak, such as medical grade face masks and hand sanitizers, to ease the panic buying. Google has introduced “Fact Check Explorer,” which verifies the information available online through authenticated third-party fact-checkers [28]. The results of a search query indicate whether the claim from each source is valid, false, partly right or partly wrong. The fact check service is available in multiple languages and is also applicable to validate an image. 

As we have mentioned above, based on the scientific literature, to date, scientists have been utilizing surveillance systems for infodemia tracking and analyzing disease outbreak-related fake news, misinformation, and rumor spread in online media and social platforms [19]. However, there is an immediate need for a tool for notifying any user about the scandal, fake news, and misinformation, while browsing in the web search engines. A web browser extension for a web search engine can be illustrated as a computer programming code package, which could be installed into a web browser that the user primarily uses for searching on the world wide web (WWW). The extension has the capability of adding a new feature to a web browser’s search engine, or augment a current functionality, update a visual theme [29], or in this case, screen a search result’s content and show warning as per severity. 

This study’s main objective was to evaluate the potential ML-based approach integrated with search engine extension for notifying any public health misinformation during this COVID-19 pandemic. This paper proposes a novel approach to prevent the spread of misinformation through a web search engine extension (SEMiNExt). Once a user inquires for specific information on the web search engine bar, SEMiNExt is activated. The extension reads the search bar’s user query and converts the texts into a number using NLP. This numerical data is then inputted into a trained ML algorithm that classifies the query’s integrity and notifies its authenticity to the user using a message box on the screen. The overall procedure transpires in real-time to alert the user of potential misinformation pitfalls before clicking on any search results. This may significantly help to prevent the spread of misinformation. 

## 2. Materials and Methods

### 2.1. Search Engine Misinformation Notifier Extension (SEMiNExt)

**Figure 1:**
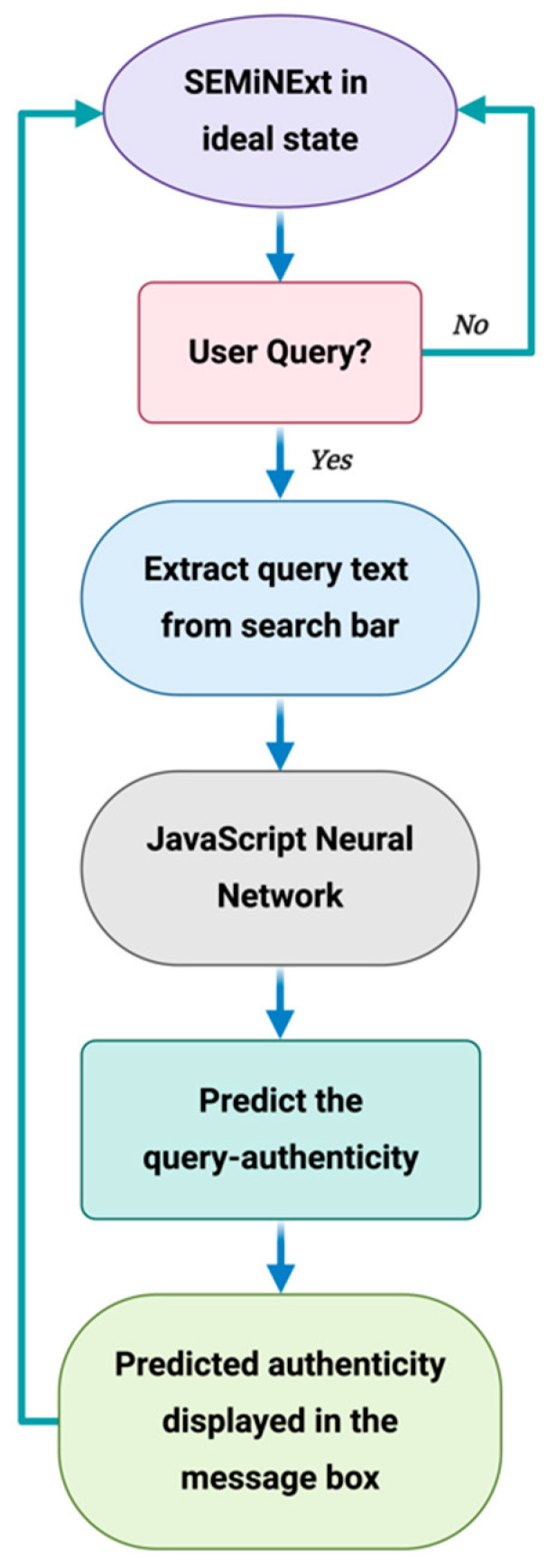
Data flow diagram of SEMiNExt. The illustration is created with Biorender.com.

The comprehensive data flow diagram of our proposed methodology is illustrated in Figure 1. Google Chrome is chosen as the benchmark web browser, as 65.47% of the netizen uses this web browser as their primary one [30]. When a user enters a text in the web search engine bar, SEMiNExt is activated and the textual content is copied and then screened for sensitive keywords related to public health. The trigger keywords for this proof-of-concept extension are—‘covid’, ‘COVID’, ‘covid19’, ‘COVID19’, ‘hydroxychloroquine’, ‘alcohol’, ‘corona’, and ‘coronavirus’. If any keyword exists, it is pre-processed further using NLP. This step is crucial to prepare the data suitable for an ML algorithm. 

After pre-processing, the data is then input into a trained ML algorithm which then predicts the authenticity of the original query. The validity of the user query is then displayed in a message box with additional recommendations to create awareness e.g., a hyperlink to visit the World Health Organization (WHO) for updated and authentic public health related information. The ML algorithm is implemented in a JavaScript Object Notation (JSON) and integrated within the extension. 

There are several machine learning steps behind the process of determining the authenticity of the query. At first, we test and compare the performances of the mentioned machine learning algorithms using python. The results show the highest accuracy for the Neural Network (NN) algorithm. Based on this, we have integrated Artificial Neural Network (ANN) into our browser extension using the built-in JavaScript focused machine learning library called Brain.js. This is a graphics processing unit (GPU) accelerated library of Neural Networks written in JavaScript for browsers. Brain.js allows training of a NN in JavaScript and then predicts based on the training outcome. The data used for training (see Section 2.2.3) has been saved in a separate JavaScript Object Notation (.json) file.

**Figure 2:**
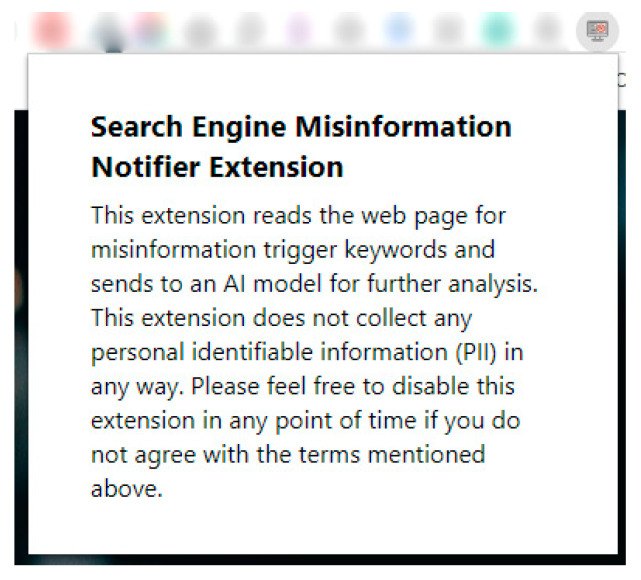
Privacy notice.

The NN in Brain.js is defined by three parameters: activation function, number of hidden layers with their respective sizes, and the learning rate. The activation function is a mathematical operation attached to each node in the network that determines the output of the NN. The function activates its corresponding node based on whether each node’s input is relevant for the prediction. The output of each node is normalized between −1 and 1. We employed the sigmoid activation function and the backpropagation method to train the nodes of our network. The learning rate determines the network tuning in response to the estimated error each time the model weights are updated. Once a user searches for anything online, a URL is generated automatically. The user query is an integral part of the URL. The extension uses the URL extractor to copy the generated URL. Then by using the content script, we can extract the query string of the user from the generated URL. When the query string has any of the trigger words, the extension Application Programming Interface (API) calls the .json file where the NN is implemented. The overall procedure transpires in real-time. The extension we developed will also work on Bing, Duckduckgo, and Yahoo! web search engines. In addition, a privacy notice is integrated with the extension (Figure 2). This informs the user about the exchange of data with the machine learning model and ensures that no personal identifiable information will be collected.

### 2.2. Natural Language Processing 

#### 2.2.1. Text Pre-Processing: 

At first, we clean all the text samples by removing any punctuation marks e.g., full stop, comma, question mark, etc. to get samples composed of only words. In the second step, all the upper-case letters are converted to their respective smaller cases. The third step involves the removal of words that are not relevant for prediction, such as “the”, “that, “in”, preposition, grammatical articles, stop words (e.g., “is” and “are”), etc. Now only the words remain that are relevant for prediction, but the inflection remains which may exist in a variety of forms either as prefixes or suffixes. To eliminate the inflections, we first split each sample sentence into its constituent words to remove the unnecessary characters, a process known as stemming. Finally, the words are stitched back to form a sentence. 

#### 2.2.2. Bag-of-Words (BoW) Model

ML algorithms only work with numbers and cannot recognize raw text. To circumvent this issue, the text must be translated into a vector of numbers that reflect various linguistic properties of the text. This is where the BoW model steps in. The idea is that texts with similar content are alike and from the content alone, we can learn about the meaning of the text. The BoW model is a text classification method where the occurrence of a word in a sample and its multiplicity are used as a feature to train a classifier. Other information such as grammar, the structure of the words, and position in a text are disregarded [31]. For an example if we have a sample: “John likes statistical tools. He likes neural network too.” it will be converted into a set of distinct words: [“John”, “likes”, “statistical”, “tools”, “He”, “neural”, “network”, “too”] and based on the frequency of the words the corresponding vector is generated: [1 2 1 1 1 1 1 1]. Once all the samples in the data set are transformed, we have a matrix of numbers that is now suitable to use in any ML algorithm. 

#### 2.2.3. Machine Learning Algorithms: 

To train various ML algorithms, we first created a data set. Each sentence of a search query is considered as a single sample and our data set holds about in total 130 samples. The samples were inspired by basic questions found in popular social media platforms such as Twitter, Facebook, and various news articles.

In our study, we have implemented the following ML algorithms on our dataset: https://github.com/ashiqur-rony/search-engine-misinformation-notifier-extension/tree/v1.0.0.

- Logistic Regression: This classifier computes the relationship between the target class (which we want to predict) and other independent variables. It is then used to estimate the probability of an outcome using a logistic function also known as sigmoid function. Therefore, the output of the function is limited between 0 and 1.
- K Nearest Neighbors (KNN): This method uses the Euclidean distance as the similarity measure. It stores all the input samples then computes the Euclidean distances of a new case relative to all the input samples. Next, K closest neighbors are used for prediction.
- Support Vector Machine (SVM): This is mostly used as a binary classifier i.e., categorize the input samples into true and false classes. At first, a mathematical hyperplane/line is introduced into the variable space. Then, the best possible coefficients are found for which the hyperplane establishes a maximum separation between the classes. The prediction is then based on the location relative to this hyperplane.
- Naive Bayes: This method computes the likelihood of every words in the sentence to appear in the true and false classes. These probabilities are used to predict the authenticity of a given sentence.
- Decision Tree: In this algorithm, a decision tree is constructed. Then based on the top-down approach, the entire input samples are searched to test every attribute at each mode. Next, entropy and information gain are calculated to identify which attribute to test at each node. This information is then used for prediction.
- Random Forest: This is an ensemble ML algorithm. Random forest combines bootstrap aggregation and random feature selection to construct a collection of decision trees to predict the final output.
- Artificial Neural Network (ANN): In this method, there is an input and an output layer. In between, there can be one or multiple layers known as hidden layers. Every layer contains at least one node and all the nodes of layer y is interconnected to every node at layer y − 1 and layer y + 1. ANN learns by adjusting the weights of every nodes iteratively via backpropagation to predict an outcome.

#### 2.2.4. K-Fold Cross-Validation 

To use an ML algorithm, we must split the input data into training and testing sets. The size of the training dataset must be maximized to achieve the best learning outcome and test data size must be optimized for an accurate prediction. More test samples mean fewer data available for the learning process and vice versa. To overcome this tradeoff, a cross-validation approach is generally employed. Cross-Validation is a statistical method of evaluating learning algorithms to estimate the error in the prediction of a model by dividing the input data into two sections: one part is used to train a model and the other is used to validate the model. In this study, we have used the K-fold cross-validation approach [32]. Here, the input dataset is randomly partitioned into K disjoint subsets (referred to as folds) of approximately equal size with no overlap between two subsets. Kohavi recommended 10-fold cross-validation for real-world data sets [33]. So, if the dataset has a total of 130 samples, then for 10-fold cross-validation, the dataset is divided into 10 equal subsets each with 13 samples. Then, 10 separate (K, in general) learning experiments are carried out. In each run, one subset is selected as the testing set, and the remaining K–1 subset is used to train the classifier. The testing set is then used to validate the trained ML algorithm. This procedure is repeated 10 times (K times in general) with 10 different testing and training sets and the results of all the experiments are averaged. In this process, the assessment of the classifier is more accurate, and all the samples are in turn used for both training and testing.

#### 2.2.5. Classification Parameters

To measure and compare the performance of various algorithms, we need to compute several parameters, namely precision, recall, accuracy, and *F*1 score. All these classification parameters can be calculated from the confusion matrix [34]. Confusion matrix is a table that visually illustrates the performance of an algorithm and provides an insight into the detail diagnosis of the model outcomes. The rows of the matrix represent the predicted classes and the columns represent the actual classes. It depicts the number and types of errors made by an algorithm. A typical confusion matrix is shown by Table 1. True Positive (TP)/True Negative (TN) are the samples that are correctly identified by the ML algorithm, and False Positive (FP)/False Negative (FN) are the samples that are incorrectly identified. These values are used to calculate the classification parameters.

Accuracy can be defined as the ratio of the correctly predicted outcomes over the total number of predictions. A drawback of this parameter is that it does not consider the subtleties of class imbalances.

(1) $$\;\mathrm{Accuracy}=\;\frac{TP+TN}{TP+TN+FP+FN}\;$$

Recall/Sensitivity is the portion of the actual positives the classifier picks up correctly among the total test data set.

(2) $$\mathrm{Recall}=\;\frac{TP}{TP+FN}$$

Precision is the portion of correct positive prediction over the total positive predictions made by the classifier.

(3) $$\mathrm{Precision}=\;\frac{TP}{TP+FP}$$

*F*1-score measures the accuracy of a model on a given dataset and is calculated as the harmonic mean of the precision and recall. It is a better measure parameter, especially in the presence of an uneven class distribution e.g., many actual negatives. An *F*1-score of 1 indicated a perfect precision and recall.

(4) $$F1=\;2\;\times \;\frac{\mathrm{Precision}\;\times \mathrm{Recall}}{\mathrm{Precision}+\mathrm{Recall}}$$

#### 2.2.6. Parameters of the Implemented Machine Learning (ML) Algorithms

For the proper outcome of the ML algorithm, we followed the parameters listed below:

- Logistic Regression.
- KNN: K = 10 nearest neighbors are used for prediction. Minkowski distance metric is used with Euclidian distance as the power parameter.
- SVM: Linear kernel is used.
- Naive Bayes: Gaussian classifier is implemented.
- Decision Tree: Entropy criterion is used in the decision tree classifier.
- Random Forest: A total number of 300 trees is used with the entropy criterion.
- ANN: Total 3 layers i.e., input, hidden and an output layers with 44, 22, 1 number of nodes respectively.

## 3. Results

### 3.1. Machine Learning (ML) Predicts Real-Time Misinformation

**Figure 3:**
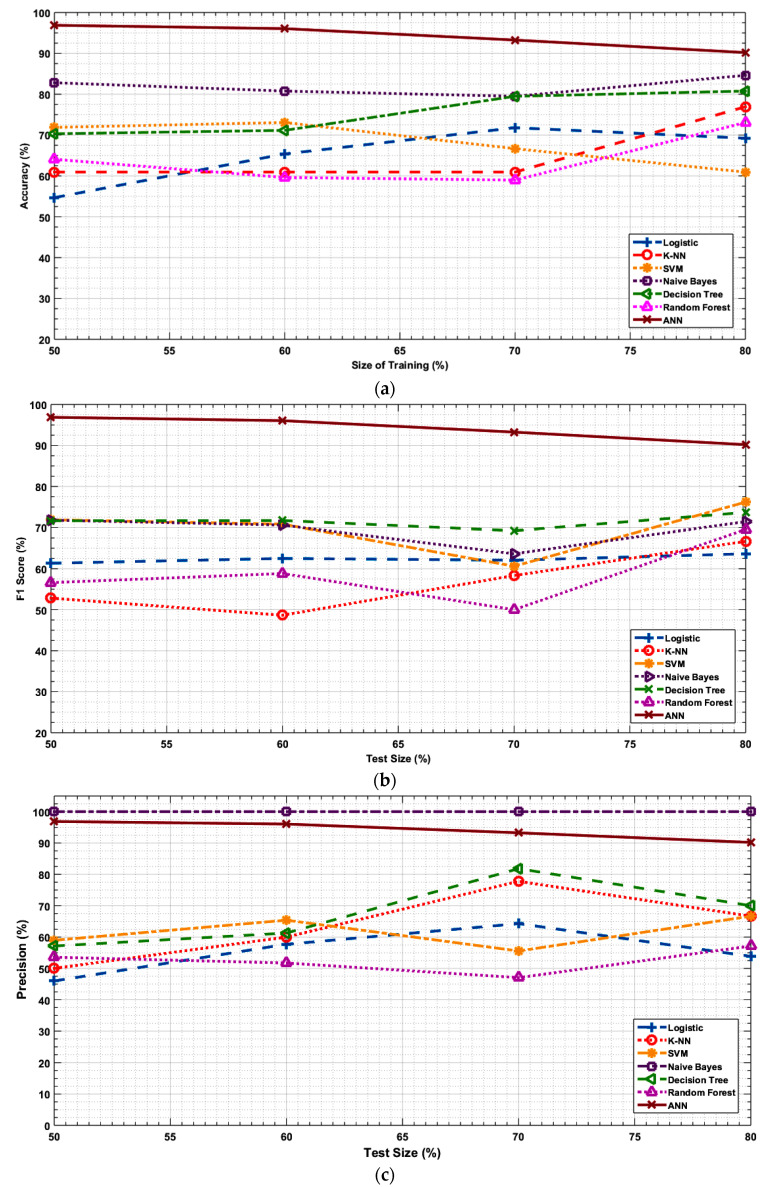

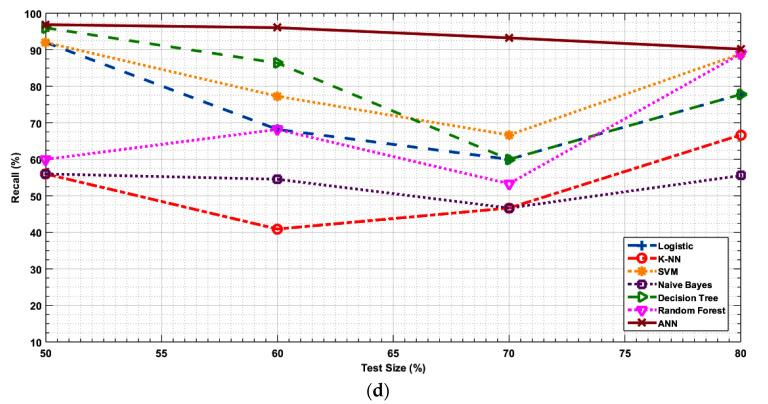
(**a**). Accuracy vs. size of the training set as a percentage of the total input data set used for training. (**b**). *F*1 score vs. size of the training set as a percentage of the total input data set used for training. (**c**). Precision vs. size of the training set as a percentage of the total input data set used for training. (**d**). Recall vs. size of the training set as a percentage of the total input data set used for training.

Figure 3a–d illustrates the accuracy, *F*1 score, precision and recall values of the ML algorithms w.r.t the size of the input data size used for training. ANN demonstrates the highest accuracy, *F*1 score and recall. Its performance decreases slightly but remains stable with a change in the data size. In general, the other statistical methods experience a slight increase in the accuracy and *F*1 score with the increase in the data size. This may be because a larger training data (pilot data set) size makes these algorithms learn and predict better. At all cases, Naïve bayes obtained a precision of 100% but it suffers from a relatively lower recall value that reduces its prediction accuracy and *F*1 score.

**Figure 4:**
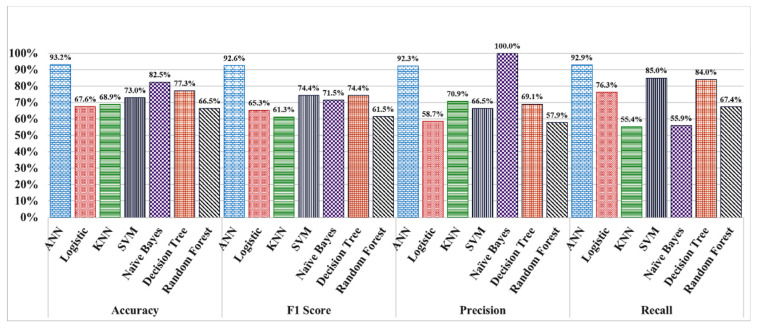
Performance comparison between the statistical methods and the artificial neural network (ANN).

The overall performances between the statistical methods and ANN is shown in Figure 4. A 10-fold cross validation was implemented to generate the results for the statistical algorithms. Since cross validation approach is not applicable for ANN algorithm, in this case, 80% of data was split for training while the remaining 20% was used for the validation. This process was repeated several times with random splitting of the input sample and the results are then averaged for a better estimation of its performance. Figure 4 illustrates that ANN achieves comparatively the highest accuracy of 93% and a *F*1 score of 92%. A high *F*1 number is important when there is an imbalanced dataset available e.g., when there are more fake news than true news. Since *F*1 score is the harmonic mean of the precision and recall, it is less affected by the extreme values. Therefore, it is a better performance indicator for an imbalanced dataset. So, ANN with the highest *F*1 score suggests that it can handle the data imbalances much better giving a more accurate prediction in classifying a given misinformation.

### 3.2. SEMiNExt Notifies Real-Time Misinformation and False Health-Related News

The SEMiNExt works as a browser extension that silently observes the search queries on different search engines. With the help of a machine learning algorithm, then it generates a message for the user to indicate the query’s authenticity. The extension is activated only when any of the predefined keywords are present in the search query.

**Figure 5:**
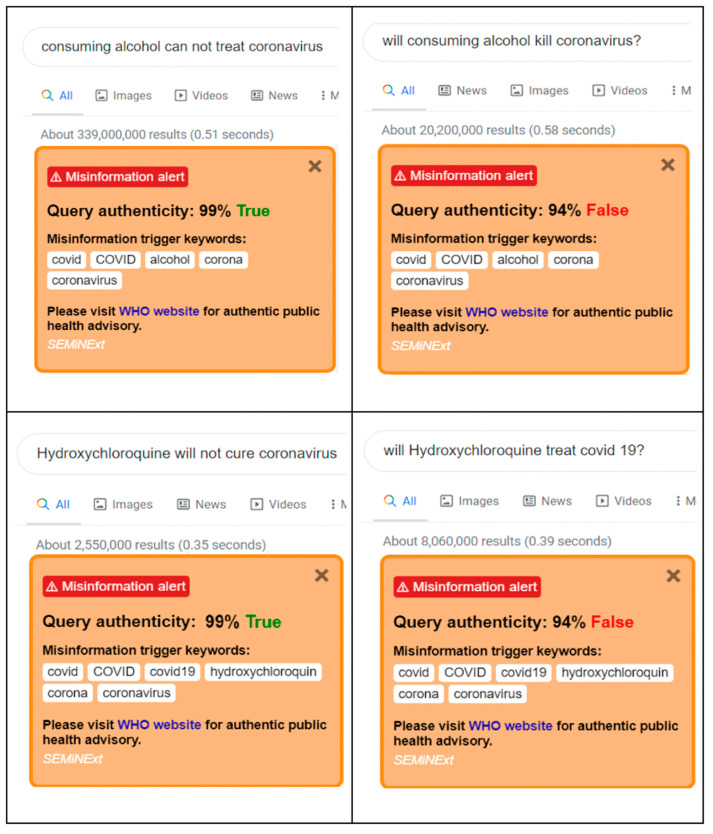
In this portion, the overall performances between the statistical methods and ANN is shown.

The trigger keywords for SEMiNExt are—‘covid’, ‘COVID’, ‘covid19’, ‘COVID19’, ‘hydroxychloroquine’, ‘alcohol’, ‘corona’, and ‘coronavirus’. When any trigger keywords are present in the user query, the extension shows a small popup at the right side of the browser with query authenticity. Additionally, the message box suggests the user to visit the WHO website through the provided hyperlink (Orange boxes in Figure 5). The proposed novel SEMiNExt works with high accuracy in the Google Chrome browser. As displayed in Figure 5, the screenshot below, a popup with the query authenticity and trigger keywords is displayed along with a hyperlink to the WHO website.

## 4. Discussion

We have proposed and demonstrated a real-time web search engine misinformation notifier extension (SEMiNExt) that uses an integrated ML algorithm to predict the accuracy of the user query. When a user inquires for a piece of health-related information in the online search engine, SEMiNExt identifies the sensitive keywords, predicts the query accuracy using ML algorithm, and triggers a message box to the user regarding the authenticity of the searched query. It also suggests that the user visit the official website of the World Health Organization via a hyperlink for raising more awareness. Our results show that SEMiNExt under Artificial Neural Network works best with an accuracy of 93%, *F*1-score of 92%, the precision of 92%, and a recall of 93% when 80% of the data is trained. Moreover, ANN can predict with very high accuracy even for a small training data size. This is very important for the early detection of new misinformation from a small data sample available online that can significantly reduce the spread of misinformation in its infancy and maximize public health safety. 

The SEMiNExt utilization will be vital for the ongoing second wave and mass panic about different COVID-19 vaccines’ updates. On 20 December 2020, the American Medical Association (AMA) has requested that social media companies combat vaccine misinformation [35]. Already the social media platform Instagram’s leading management said months ago that they would take measures to slow down the spread of any anti-COVID vaccine-related misinformation and fake news. However, several vaccine-related contents were reported by different news agencies [36]. Surprisingly, it has increased to spread virally in the Instagram platform, which created a lot of alarm for the public health officials and government authorities [37].

Therefore, our proposed novel protocol can be utilized as a useful tool for obstructing the spread of new circulating false information. We can observe from Figure 4 that unlike the statistical methods, ANN can predict with relatively very high accuracy even for the availability of smaller data size. This is very important, especially in predicting new misinformation that started making its rounds online very recently and has minimal related data available online. Therefore, using ANN, the early detection of potentially new rumors from a small data sample can is possible with high accuracy. In this regard, SEMiNExt can intervene and significantly reduce the spread of misinformation and maximize public health safety before the fake news escalates into a significant panic amongst the population.

The current pandemic has caused hospital ICUs and emergency departments to become overwhelmed by untenable patient volumes and care requirements [38]. Any novel viruses cause intense anxiety among people searching for a rapid cure. In this circumstance, false treatments may appear online, encouraging someone to try it against scientific validations. Such extra patients can quickly saturate hospital beds and other medical equipment during an ongoing pandemic. When searching for cures online against coronavirus, SEMiNExt validates the user query in real-time to warn the user against the pitfalls of such false treatments. This can prevent the overload of hospitalizations from non-COVID patients. It would also alert the healthcare authorities about the potential regions of heavy misinformation related online searches and alarm the local authorities to take measures, even advising the hospitals about any rush in the emergency. 

## 5. Conclusions

We have mentioned earlier that during any disease outbreak, the health care system faces massive challenges for online misinformation and fake news. SEMiNExt can successfully prevent the depletion of healthcare resources: in novel disease outbreaks, resource management in healthcare can be overwhelming because the demand is dynamic. Our proposed method can be extended to real-time healthcare resource management. At the onset of the COVID-19 pandemic, a strict lockdown was implemented to slow down the virus’s widespread. This caused anxiety amongst the general population about food availability, medical resources, and other necessities over the lockdown period. As a result, a private accumulation of common medical resources such as medical masks, oxygen cylinders, etc., was observed. Later, a shortage of resources was experienced in many hospitals when the number of critical patients started rising exponentially. This was a familiar scenario in most of the developing countries. When inquiring online about the essential medicines or medical equipment that are more critical to the hospitals, SEMiNExt can inform and discourage the user against a private stockpile. This can ensure that the medical inventories are never understocked with healthcare resources. 

The SEMiNExt approach has introduced the possibility of improving the online health communication system by showing misinformation notifications in real-time, enabling safer web-based searching while inquiring on health-related issues. SEMiNExt is designed to function properly in multiple web search engines platforms, such as Google, Bing, Duckduckgo, and Yahoo. Additionally, in this study, only English has been chosen as the natural language; additional natural languages, such as (by the descending order, according to the speaking population) Spanish, Bengali, Russian, German, French, and Italian, will be integrated to the extension in the future.

## Figures and Tables

**Table 1:** Example of a confusion matrix.

Predicted\Actual	Positive	Negative
**Positive**	True Positive (TP)	False Positive (FP)
**Negative**	False Negative (FN)	True Negative (TN)

## Data Availability

The novel search engine misinformation notifier extension (SEMiNExt) introduced and presented in this original paper have been stored in the GitHub repository: https://github.com/ashiqur-rony/search-engine-misinformation-notifier-extension/tree/v1.0.0.
